# Modeling IoT-Based Solutions Using Human-Centric Wireless Sensor Networks

**DOI:** 10.3390/s140915687

**Published:** 2014-08-25

**Authors:** Álvaro Monares, Sergio F. Ochoa, Rodrigo Santos, Javier Orozco, Roc Meseguer

**Affiliations:** 1. Department of Computer Science, Universidad de Chile, Beauchef 851, Santiago 8370459, Chile; E-Mail: amonares@dcc.uchile.cl; 2. Department of Electrical Engineering and Computers, IIIE, UNS-CONICET, Alem 1253, Bahía Blanca 8000, Argentina; E-Mails: ierms@criba.edu.ar (R.S.); jorozco@uns.edu.ar (J.O.); 3. Department of Computer Architecture, Universitat Politècnica de Catalunya, Jordi Girona Salgado 1, Barcelona 08034, Spain; E-Mail: meseguer@ac.upc.edu

**Keywords:** Internet of Things, modeling approach, human-centric wireless sensor networks, emergency response, urban emergencies, ambient intelligence, information sharing

## Abstract

The Internet of Things (IoT) has inspired solutions that are already available for addressing problems in various application scenarios, such as healthcare, security, emergency support and tourism. However, there is no clear approach to modeling these systems and envisioning their capabilities at the design time. Therefore, the process of designing these systems is *ad hoc* and its real impact is evaluated once the solution is already implemented, which is risky and expensive. This paper proposes a modeling approach that uses human-centric wireless sensor networks to specify and evaluate models of IoT-based systems at the time of design, avoiding the need to spend time and effort on early implementations of immature designs. It allows designers to focus on the system design, leaving the implementation decisions for a next phase. The article illustrates the usefulness of this proposal through a running example, showing the design of an IoT-based solution to support the first responses during medium-sized or large urban incidents. The case study used in the proposal evaluation is based on a real train crash. The proposed modeling approach can be used to design IoT-based systems for other application scenarios, e.g., to support security operatives or monitor chronic patients in their homes.

## 1. Introduction

The Internet of Things is probably one of the most challenging and disruptive concepts raised in recent years [1,2]. It pushes systems designers to address complex aspects that are recurrent in these solutions, for instance, the heterogeneity of devices and communication interfaces that are present in the working environment, the devices' mobility and computing capability, and the interoperability of data and services. Usually a device participating in an IoT-based solution (e.g., a surveillance video-camera) could also be part of more than one solution. For instance, it can assist the security system and also the automatic fire detection systems. Although this multi-role participation of devices is highly recommended, it increases the challenges for designing IoT-based solutions.

Several researchers have highlighted the need to find an agreement on the IoT concept and provide guidelines and tools to help address the design of these solutions [3]. In that sense, some reference architectures have been proposed to structure these solutions [4,5], and also some general design guidelines have been presented [6]. However, there is no clear approach to modeling the interactions among the participating devices, allowing designers to evaluate the system behavior before implementing it. Therefore, developers must conceive their solutions using only their expertise. This design approach is risky since the IoT-based systems belong to a new development area where not much expertise has been accumulated and shared. If the system being modelled has to support a critical activity, such as military operations or emergency responses, the designers must know and inform the limitations of the solution. A late detection of these limitations (e.g., while the system is being used in a real scenario) could produce serious drawbacks to the process supported by the application.

Dealing with that challenge, this paper proposes a modeling approach that uses a human-centric wireless sensor network (HWSN) [4] not only to design IoT-based systems, but also to identify their potential limitations before implementing them. This approach allows designers to assign a basic role to the components participating in an IoT-based solution and characterize the communication links between them. Thus, it is possible to evaluate, at the design time, the information generation and flow through the whole system. Designers can choose to address or assume the system limitations, but it will always be made in an informed way.

This modeling approach supports the evolving development of systems designs, allowing the implementation to be done once most system behavior has been already verified. The use of this approach should contribute to reducing development risks and costs. This proposal does not address the system implementation, but it allows considering the features of the devices and communication links used to implement the solution. Therefore, we can consider this modeling approach as complimentary to the existing solutions for implementing IoT-based systems. Particularly, the nodes features and capabilities, and also the interaction protocols among them, can be set during the pre-design phase; *i.e.*, when the designer defines the types of nodes and links that will be present in the new system model.

Transversal design aspects, such as information security and privacy, are not formally considered in these models. However, these aspects can be addressed as capabilities related to the roles played by the participating nodes, as suggested by Sicari *et al.* [7]. An interesting component that helps deal with information trustworthiness, security and privacy in these networks is the nodes position, as indicated in Coen-Porisini *et al.* [8] and Basilico *et al.* [9]. Nodes' reputation [10] can also be used to address information trustworthiness and data fusion.

The article also shows how interaction scenarios can be modelled based on nodes of different types, with certain characteristics, which can then be simulated to find the best solution for a scenario, without actually implementing it. In order to illustrate this capability, we present the design of a hypothetical IoT-based solution that supports medium-sized and large urban emergency response processes. The expected behavior of the system is compared with the real response process performed by first responders (*i.e.*, firefighters, police officers, paramedics, and emergency managers) after a train crash happened in Buenos Aires (Argentina), on 22 February 2012. A possible implementation of the hypothetical IoT-based solution is shown and evaluated using a network simulator, only to illustrate the usefulness of using HWSN to modeling these systems.

Next section briefly describes the regular process to address medium-sized or large urban emergencies. Section 3 discusses the related work. Section 4 presents the foundations of the HWSN. Section 5 describes a general architecture that can be used to arrange the components that are present in a HWSN. Section 6 exemplifies, using a real urban emergency situation, how this hypothetical supporting system could have contributed to reducing the incident impact after a train crash. We also discuss how to model and evaluate this system before implementing it. Section 7 presents the conclusions and future work.

## 2. Urban Emergency Responses

Medium-sized and large emergencies affecting urban areas (e.g., train derailments, fires affecting buildings, and accidental or intentional explosions) usually represent a challenging situation for first responders, because of the lack of event early detection systems, mechanisms to perform a quick diagnosis of the situation, and supporting information to make decisions in a distributed way. The complexity of the urban scenarios usually increases this challenge.

Immediately after an incident occurs, a 911 service receives the emergency calls. The operators then have to determine the veracity of such an incident, and perform a first diagnose of it (type, size and complexity) using the information provided by the people [11]. Based on this diagnosis, the operators dispatch resources to perform the first response process. The diagnosis and dispatching processes usually take some minutes.

These resources have to arrive quickly to the emergency place since the reaction time and the efficiency in the first response are crucial to reducing the number of victims in an emergency [11,12]. In [13] it has been proven that reducing the first response time by one minute increases a six percent the number of lives saved in car crashes. These numbers are probably representative of other urban incidents.

The coordination of the emergency response activities represents a major challenge to first responders for several reasons: (1) civilians usually go to the affected area to see what is happening, obstructing thus the transportation of resources and the response process; (2) services in the area (e.g., mainly communication and transportation) tend to collapse or they are temporarily suspended for safety reasons (e.g., energy, gas and water); (3) the available radio channels used by first responders are not enough to coordinate the response activities; and (4) there is little or no supporting information to make decisions and coordinate the efforts among the participants. Therefore, improvisation is the regular pattern in these situations [14,15].

Clearly, supporting these emergency response activities is a huge challenge, and there is probably no system able to deal with it. However, we can address part of it using an IoT-based solution; particularly the communication support and the information flow can be improved using this type of system. The following section presents different approaches and proposals that can be used or adapted to deal with such a challenge.

## 3. Related Work

The use of mobile devices, wireless sensor networks, and even the use of IoT-based systems to support urban first responses has been addressed by several researchers. Concerning incident detection, White *et al.* [16] proposed an automatic mechanism for traffic accident detection and notification using smartphones. It delivers early alerts to particular emergency centers and thus accelerates the response process. Similarly, Liu *et al.* [17] use cyber-physical elements (called intelligent guards) deployed in the physical infrastructure to deliver early alarms when an extreme event affects a certain urban area.

Concerning the support for the first response process, Martin-Campillo *et al.* [18] propose a RFID-based solution to tag injured people, indicating their health condition before delivering them to a hospital. Several researchers propose the use of mobile ad hoc networks, usually implemented using WiFi, to provide communications support in disaster areas [17,19,20]. Ochoa and Santos [4] go a step further and introduce the concept of human-centric wireless sensor networks. They also show how HWSN-based solutions can be used to increase the information availability in the affected area, and thus allow first responders to make informed decisions.

Zhang *et al.* [21] describes an IoT-inspired platform, which was designed to support emergency management systems. Although such a proposal is quite general, it allows us to see how the several components participating in the process can interact with one another to capture or disseminate shared information.

Yang *et al.* [22] propose a modified “task-technology fit” approach to help developers understand how the IoT technology can enhance the urban first response activities. This approach does not indicate how to design IoT solutions to support these operations, but it allows us to carry out the role of IoT technology in urban emergencies.

Roggen *et al.* [23] propose an opportunistic human activity context recognition based on already deployed sensors, taking advantage of the increasing connectivity provided by these sensors and the IoT capability to support true ambient intelligent applications.

In [24] the authors introduce a data management solution for IoT systems that are capable of generating a very large amount of data. The proposal follows a distributed data- and sources-centric approach.

Lee *et al.* [25] introduce a different approach to design user-centered IoT systems. The authors argue that a bidirectional processing mechanism between internal knowledge representation (human knowledge) and external knowledge representation (networks of objects), plus an uncertainty-driven arbitration mechanism that acts on this knowledge base, are foundational to the design of truly user-centered IoT systems. These computational principles, underpinned by an enriched understanding of the psychology and neuroscience of human learning, will significantly improve the user's experience in a variety of IoT scenarios.

The IoT is becoming a challenging subject for researchers all over the world, as it provides infinite alternatives for developing applications for acquiring, processing and distributing data, information and knowledge. In [26] the authors introduce a new approach to integrate IoT and software-as-a-service (SaaS) model for logistic systems. They propose a framework to design logistic systems based on SaaS cloud computing and IoT technology that uses the cloud.

Several initiatives in the European Union and the United States work towards device integration to support early detection of urban emergencies. For instance, Sensors ANYware (SANY) [27] is an initiative oriented to obtain an open standard for integrating already deployed sensor networks with those to be deployed in the future. Similarly, the Integrated Public Alert and Warning System (IPAWS-Open) [28] provides services to receive, authenticate, and route standards-based messages from alerting authorities to all types of alert systems, via several communication pathways, including public broadcast, cellular networks and the Internet.

In [29] the authors introduce an emergency fire management system in the Web of Object infrastructure. They integrate the formation and management of Virtual Objects (ViOs), which are derived from real world physical objects and virtually connected to each other in the semantic ontology model. This allows information reusability, extensibility and interoperability, which enable ViOs to uphold resource orchestration, federation and collaboration. All these aspects are necessary for the coordination of actions in the case of fires.

Although the previous proposals are interesting and contribute to improving the effectiveness of the first response process, they are focused on addressing a particular aspect of the problem. Moreover, none of them allow designers to represent the type of components that participate in an IoT-based solution, the role played by each of component, and the way in which the shared information is captured and disseminated through these networked solutions.

In the next section we present the basic concepts behind a Human-centric Wireless Sensor Network (HWSN) [4], which allows modeling the nodes interaction and the information flow in IoT-based systems. Moreover, we indicate how these concepts can be used to determine the components roles, their capabilities for information processing and communication. As mentioned before, there are various transversal design issues, such as information trustworthiness, security and privacy that do not have been formally considered in the model of a HWSN. However, these design aspects can be included in the network model through the use of complimentary elements, such as roles [7], location [8,9] and reputation of the nodes [10].

## 4. Foundations of HWSNs

A HWSN is a conceptual network that supports processes being performed in a physical fashion; for instance the search-and-rescue process performed by firefighters during large fires or after a building collapse. This network represents a bridge between the physical and the digital worlds. The nodes participating in a HWSN can consume, deliver, store and process information, and also interact with the real world, e.g., delivering visual or sound messages.

Each network node (e.g., a smartphone or a surveillance camera) has at least a communication interface that allows it to interact with other nodes. The interaction between nodes is based on message passing. According to the definition presented in Equation (1), each message must count on at least the following attributes:

(1) message∷=<sender,destination,timestamp,timeout,information>

where the timestamp indicates the message delivery time and the timeout is the message expiration time. The information held in the message is relevant only for the destination node and such content is processed at the application level; therefore the information piece must have at least the following attributes:

(2) information∷=<source,timestamp,type,weight,data>

where source indicates the user (and/or the role) that is sharing the piece of information (typically it is the owner) and the timestamp indicates when this particular sharing process was triggered. This information is typically used to determine the age or validity of the information piece. The information type represents meta-information that can be used by the destination node to decide how to process such data. For instance, this attribute can indicate if the data is a video, text, image or a resource with a particular data structure (e.g., a personal record). The information weight is also meta-information that is used by some nodes to decide how to perform the role assigned to them. For instance, processing or transferring heavy-weight resources could take long time; therefore the node could prioritize to serve other low-demand requests. Next we describe the roles that can be played by the network nodes.

### 4.1. Nodes Basic Roles

In a broad sense, the concept of node in a HWSN is the same as with almost any communication network; *i.e.*, a physical device that interacts with others through a communication interface. However, these nodes can play one or more roles. Every role can be seen as a function or process with an input and an output data interface, as shown in Equation (3). Typically, the process receives the data input, processes it and produces an output; *i.e.*, similar to a filter in the pipe-and-filters architectural pattern [30]:

(3) role∷=<input,process,output>

If a node plays more than one role, the services provided through these roles can be played in an independent way, or they can be organized through a pipeline or workflow. For instance, a smartphone is a node that can play the role of “sensor” since it can capture images using its camera. It can also play the role of “processor” because it has computing capability of performing specific tasks. These services can be provided independently of one another. However, if the smartphone should provide a people presence awareness service (*i.e.*, a complex service), the system must connect these two roles in a workflow indicating that such a service considers two steps: capturing a picture and trying to identify known people in it through face recognition as shown in the following services definitions:

(4) presence_awareness∷=<s_picture_capture|p_face_recognition>

(5) s_picture_capture∷=<null,take_picture_pa,picture>

(6) p_face_recognition∷=<picture,picture_recognition_pa,bool>

The s_picture_capture service does not have a formal input and the output is a picture as defined in Equation (5); the prefix “s_” indicates that such an operation is a sensing activity (*i.e.*, a role played by the node). The p_face_recognition service takes the output of the previous service and delivers a true or false depending on the results of the recognition process as defined in Equation (6). The prefix “p_” indicates that such a service is a processing operation (*i.e.*, other role). If the operation specified in the definition does not have a prefix, it means that it is atomic and has a particular implementation on the device performing the action.

**Figure 1.**
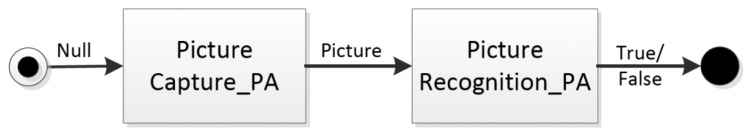
Structure of the presence awareness service.

Provided that every role is a process that can be seen as a filter in a pipeline (Figure 1), both the input and the output for each filter can be active or passive. A filter with an active input means that such a process is aware of the arrival of an input data to process it. Contrarily, the input is passive if the process is triggered only after a direct invocation done by the next filter in the sequence or by an external source. Something similar happens with the outputs of each filter. A filter with an active output delivers the process results to the next filter in the pipeline or to the target entity. However, if the output is passive, the filter stores the result in a buffer or keeps it until the next filter in the pipeline asks for such data. Clearly, the information flow in these structures depends on properly coordinating the input/output behavior of the filters in the pipeline.

In our example, the output of the first filter is active and the input of the second one is passive; it could also be in vice versa. In another case, the information does not flow between filters or can produce information collision. The suffix in the operation name indicates the behavior that the filter adopts when processing an input and an output; *i.e.*, the suffix “_pa” shown in Equations (5) and (6), and also in Figure 1 indicates a passive behavior for the filters. The behavior of complex services, *i.e.*, those created through the integration of other services is automatically determined by the behavior of the first and the last filters in the workflow; therefore these attributes do not need to be specified for these processes. These properties of the filters also determine the level of coupling between them.

The HWSNs consider six basic types of roles for the nodes, and also any composition among them. Next, we explain each role.

### Regular Sensor (RS)

This role allows a node to measure a particular variable on-demand. The measured variable can be continuous or discrete. A smartphone can play this role if its peripherals are used to capture audio or video (*i.e.*, sensing) of the environment. This role can also be played by a weather station that informs on-demand the wind speed and direction to software applications. This role considers only two actions (or services) that are performed in a sequential way, as defined in Equation (7): the reception of an order indicating the measurement request (8), and the delivery of the measurement results (9). Therefore we can expect that a regular sensor has a passive input and an active output. However, a sensor can simulate an active behavior if we add a processing unit that controls the regular sensor. Nodes playing only this role have no capability to detect other nodes in the HWSN:

(7) regular_sensor∷=<retrieve_request|deliver_result>

(8) retrieve_request∷=<message,gather_request_pa,return_request>

(9) deliver_result∷=<request,measure_variable_pa|return_result>

### Human Input Mechanisms (HIM)

**Figure 2.**
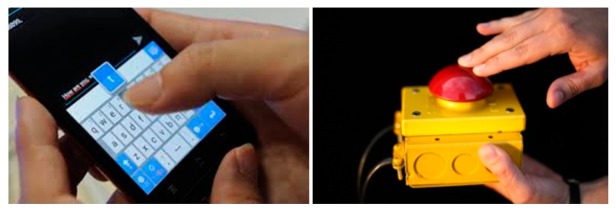
Examples of human input mechanisms.

This is another mechanism to input information to the HWSN. Devices playing this role allow people to add information or trigger commands through the network. Several peripherals (e.g., a virtual or physical keyboard) or devices (e.g., a pushing button) can play this role (Figure 2). In this case, the data input is performed on-demand (*i.e.*, the input to this filter is passive). Using human inputs the user can also trigger an alarm or make a request to other nodes, e.g., to ask for information about a situation or a process that is being managed through the network.

As shown in Equation (10), this role performs one action and it does not receive information from other nodes; only a person (or robot) can invoke the service provided by this role. Once received an invocation, the service receives the input (data or command) and deliver it through the network:

(10) human_input_mechanism∷=<invocation,data_gathering_pa,message>

#### Witness Unit (WU)

This role provides two independent services: storing and retrieving shared information on-demand, as shown in Equation (11). This role follows a producer/consumer paradigm and it considers a limited storage capability for the nodes. Typically, servers or public temporal repositories play this role. Mobile computing devices could also play such a role for short time periods:

(11) witness_unit∷=<storing_information or retrieving_information>

(12) storing_information∷=<message,processing_storing_request_pa,bool>

(13) $$\begin{array}{l} \text{processing}\_\text{storing}\_\text{request}\_\text{pa}∷=\text{if}<\text{storage}\_\text{capacity is not full}>\text{then}\\ <\text{retrieve}\_\text{information}|\text{store}\_\text{information}\\ |\;\text{return}\_\text{true}>\\ \text{else}\\ \text{if}<\text{information}\_\text{to}\_\text{replace is not null}>\text{then}\\ <\text{retrieve}\_\text{information}|\text{store}\_\text{information}\\ |\;\text{return}\_\text{true}>\\ \text{else}\\ <\text{return}\_\text{false}> \end{array}$$

(14) $$\begin{array}{l} \text{retrieving}\_\text{information}∷=<\text{message},\text{processing}\_\text{retrieval}\_\text{request}\_\text{pa},\\ \text{message}> \end{array}$$

(15) $$\begin{array}{l} \text{pocessing}\_\text{retrival}\_\text{request}\_\text{pa}∷=\text{if}<\text{information}\_\text{available}>\text{then}\\ <\text{retrieve}\_\text{information}|\text{create}\_\text{message}\\ |\;\text{return}\_\text{message}>\\ \text{else}\\ <\text{return}\_\text{message}\_\text{null}> \end{array}$$

The storing_information service is performed when a node asks to a Witness Unit (WU) for storing a piece of shared information. Such a request is done through a message as defined in Equation (12). In order to determine if it is possible to store that information piece, the WU analyzes its current content to determine if there is available space or piece of information that can be removed because it has lower priority. In both cases the WU retrieves the information from the message, stores such information and indicates that this operation was successful Equation (13). In another case, the WU informs the requester that the operation failed, because the information stored in the WU had at least the same priority as the piece sent in the request. The priority of the information is controlled by *resolutors* that work similarly to those proposed in [31]. The retrieving_information service is also invoked on-demand through a message that is sent to a WU. The message indicates which piece of information is required. The WU search for such a piece in its local repository, and if it is found, it is returned to the requester through a message (14). In other case, a message_null is returned (15) indicating that the piece of information was not found.

#### Mule (Mu)

In contrast to the WUs that are information holders, the mules are messages holders. These mules are usually mobile nodes that contribute to routing messages from a source to a destination node. Typically, vehicles having a computing device (e.g., ambulances or fire trucks) can play this role. In many work scenarios, where the communication links collapse (for instance, in urban emergency responses), the use of mules is vital for sharing information among the participating units. Similar to the WU, the mules have a limited space to store messages; therefore they have to manage that space using resolutors. Nodes playing the role of mule perform three activities (or services): receive messages, detect the presence of the nodes in the environment, and deliver messages as shown in Equation (16). These activities are loosely-coupled and it is not for sure that all of them can be completed by the mule; therefore we consider these services as independent. The storing_message service is similar to Equation (12) but it uses *messages* instead of *information*:

(16) mule∷=<storing_messages or sensing_environment or delivering_message>

(17) sensing_environment∷=<message_target,detecting_node_aa,bool>

(18) delivering_message∷=<target_node,delivering_message_aa,bool>

(19) $$\begin{array}{l} \text{delivering}\_\text{message}\_\text{aa}∷=\text{if}<\text{contact}\_\text{period is enough}>\text{then}\\ <\text{deliver}\_\text{message}|\text{return}\_\text{true}>\\ \text{else}\\ <\text{return}\_\text{false}> \end{array}$$

The sensing_environment service is used by the mules to detect the nodes that are target of the messages held by these transporters. Therefore the mules receive a message_target, which can be a physical node or a user role. Based on such information it senses the environment and tries to detect the destination node (17). If the detection process is successful, then the service returns a “true”. Depending on contact_period of the mule, the message is delivered or not Equation (19). The idea behind such a decision is to deliver a message only if the mule is sure that there is enough time to transfer it completely, in another case the transfer intend would be a waste of time and energy, and it would jeopardize the delivery of other messages. These mules can be periodic or random. Periodic mules move through a specific route, making stops in predefined places. The speed of these mules between two points can be estimated, as indicated in [4]. However, the random mules are typically vehicles that support a particular activity (e.g., an ambulance) and therefore they do not follow a route or have predefined point to collect and deliver messages. In this case the mule service is provided while the vehicle supports its main activity (e.g., transporting injured people to the hospital).

#### Processing Unit (PU)

This role can be played by devices having computing capabilities, and it considers receiving information from other nodes, processing it and then returning it accordingly. Typically, these services interpret, transform or fuse information to generate new knowledge that is usually shared through the network. These units can be active (if they autonomously process the information) or passive (if they need to be invoked by others services). The definition of both unit types is similar. In Equation (20) we present the definition of an active processing unit:

(20) $$\begin{array}{l} \text{processing}\_\text{unit}∷=<\text{information},\text{information}\_\text{processing}\_\text{aa},\\ \text{return}\_\text{information or command}> \end{array}$$

The information_processing_aa service provides ad hoc functionality that depends on the specific information processing that the node have to do; for instance to recognize a face in a picture. It is assumed that a processing unit counts on the memory and storage enough to perform the processing activity assigned to it. These units can also make decisions as part of the processing activity, and therefore the output can also be a command. For instance, if a processing_unit determines that the quality of the air in a room has fallen below a certain limit, it can trigger a command to an actuator indicating it to open a window.

#### Actuator (Ac)

**Figure 3.**
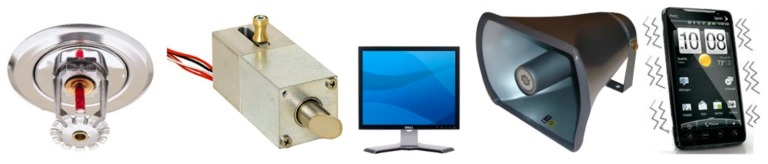
Examples of actuators.

This role represents the output of the system. There are several types of components to produce these outputs, as shown in Figure 3. For instance, a sprinkler or an electronic lock produces a physical action when they are activated. A device screen produces a visual output, while the horn or the vibration system of the smartphones generates sound and tactile stimulus respectively. These outputs usually provide feedback to the ubiquitous system users, who can eventually use a human input mechanism to add new information to the network.

Typically, the actuators are controlled by a processing_unit, which uses them to impact the physical world, including people. Therefore, the actuators receive a command from one node (e.g., from the virtual world), and translate it in an equivalent stimulus that is deployed in the physical world, as shown in Equation (21). The implementation of the generate_output_pa service is ad hoc, since it depends on the output mechanism and the information to be deployed:

(21) actuator∷=<command,generate_output_pa,null>

### 4.2. Nodes' Complex Roles

According to the previous definitions, there are three basic roles that allow adding information to the network: the regular sensors, the human inputs and the information processing units. The outputs can only be provided through the actuators, and the storage needs are addressed by mules and witness units. Using these roles we can model a large amount of IoT-based systems and also complex roles for the nodes. For instance a car alarm can be modeled as composed of a human input (the remote control), an information processing unit (the integrated system managing the alarm signals) and an actuator (the horn).

An example of a complex node is a smartphone. It has several regular sensors (e.g., accelerometer, GPS and microphone), human inputs (e.g., keyboard or microphone) and actuators (e.g., speaker, vibration system and screen). The smartphone also embeds a processing unit and has storage capabilities. This simple example illustrates the mix of roles and the complexity that can be available through the nodes.

**Figure 4.**
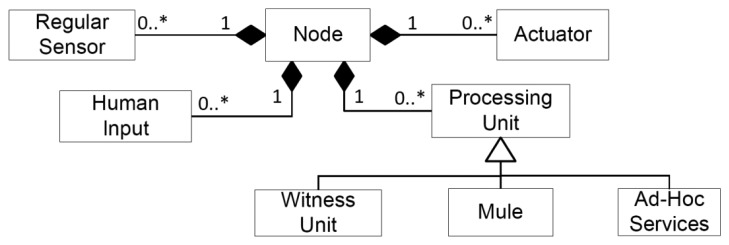
Roles composition for a node.

A complex node that must be present in any HWSN is the Human-Based Sensor (HBS) [4]. The HBS can retrieve information from other sensors and HBS, perform information fusion and feed the network with new information pieces. Typically, it is implemented as a mobile software application used by a person; therefore, the conjunction of the device, the software running on it and the person operating the application represents the HBS. The capabilities of the HBS assume that these nodes should have at least one human input mechanism and one actuator. Figure 4 shows the way in which the node roles can be composed to create complex nodes.

The actuator, regular sensor, human input and processing unit are independent roles; therefore they can be assumed by the node without restrictions. However, the witness units and the mules require having a processing unit to provide their services. The processing units can also implement ad-hoc services that depend on the job performed by the unit.

The mules interact with other nodes through message exchange, as defined in Equation (16). However, the processing units interact with others using information pieces or command, as defined in Equation (20). Typically these units expose or consume services (e.g., Web services) from other nodes.

### 4.3. Communication Links

As previously mentioned, each node in a HWSN has at least one data communication interface and it is able to process network messages. The capabilities of a communication interface depend on the technology used to implement it (e.g., WiFi, Bluetooth, infrared or 3G). Particularly, the use of a specific technology determines the communication threshold between two network nodes, the bandwidth of the communication channel and the reliability of the link between nodes. The reliability of the link also depends on the demand for message transportation received by the channel. For instance, the communication requests after a disaster (e.g., an earthquake) surpass largely the bandwidth available in cellular networks. Thus, a regularly reliable network is rendered unreliable for a certain time period.

In HWSN we assume that the link between two nodes can be classified into: *stable*, *unstable* and *null*. The link is stable (*i.e.*, reliable) if there is a high probability (over 2/3) that two nodes can communicate between each other when required. Reliable links must be implemented among nodes that need to communicate in a rapid manner or that work in a tightly-coupled way; for instance, between a sensor that detects smoke and the processing unit that activates the sprinklers.

Unstable reliability means that the probability for effective communication between two nodes is between 1/3 and 2/3.This communication should be provided between nodes that exchange non-sensitive or critical information. The coupling among nodes using an unstable link should be loosely-coupling; for instance a node that needs the help of a mule to disseminate a low-priority message.

Finally, the link will be null if there is not a direct link between the nodes that need to interact or if the probability to have an effective communication is low (e.g., below 1/3). In this case, the nodes could interact using intermediary nodes, for instance through a mule. This segmentation of quality links is inspired by recommendations made for managing the communication links in groupware applications [32].

**Figure 5.**
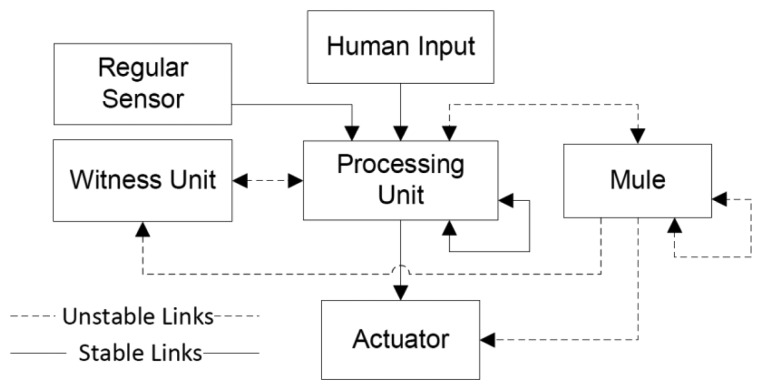
Expected quality links according the node role.

The use of routing strategies in this scenario should be analyzed carefully, because it could result in more drawbacks than advantages. For instance, the energy consumption of the nodes and also the number of messages in the network can rise considerably, generating thus an early collapse of the supporting network. The information sharing between these nodes can be done using witness units. Figure 5 illustrates the type of interaction that is usually present among nodes that play different roles.

The interaction based on information tends to be more coupled than the interaction based on messages, therefore the communication links between nodes exchanging information (e.g., the components of an HBS: sensor, human-input, processing unit and actuator) should be as stable as possible. The interaction with mules or witness units is loosely-coupled, therefore the communication link could become unreliable. Of course, the higher the reliability of the communication links, the faster the communication process. Designers of IoT-based systems should try to have links as reliable as possible; however in several scenarios, like in disaster relief efforts, having a stable link is almost impossible [4,15]. The temporal instability of the link could be addressed using multi-hop communication, as proposed in [33].

Estimating the quality of the communication links that would be available in the field will allow designers to determine several features of the solution; for instance: how fast the information can be disseminated through the network, which nodes are causing a bottleneck, or what percentage of nodes can receive a piece of information in a certain time period. Based on these results, the design of the system can be improved in an evolving way during the design time. Thus, the system can be implemented once its features have been determined analyzing the capabilities of the system model. Thus, the use of this modeling approach helps reduce development times, costs and risks.

## 5. Architecture of an IoT-Based Solution

**Figure 6.**
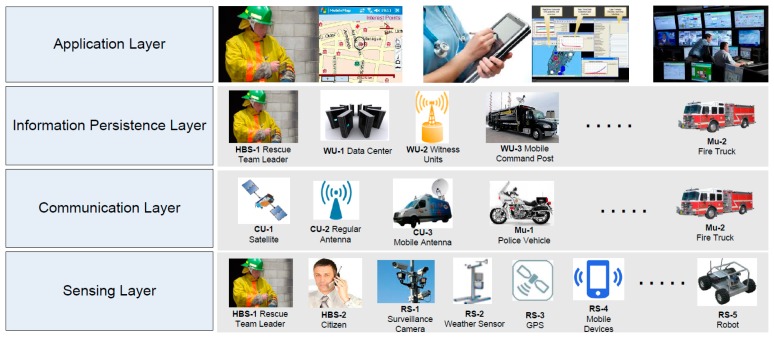
Hierarchy of architectural components.

Internet of Things infrastructures allow data and services integration among smart objects (e.g., mobile robots), sensing devices and human beings, using different but interoperable communication protocols [34]. Following this definition, Figure 6 shows the architecture proposed for systems that support first responders in medium-sized or large urban incidents. The architecture involves four layers that implement the separation of concerns: *sensing*, *communication*, *information persistence and application* (*i.e.*, usage).

The *sensing layer* is responsible for capturing information from the field, which will be then used to support the decision making and coordination activities. Two types of components contribute to performing this activity: *regular sensors* (RS) and *human-based sensors* (HBS). The first ones (e.g., weather and motion sensors, or video-camera) capture information from the environment and transmit it through a component of the communication layer. The HBS (typically firefighters, police officers or government personnel) perform the same activity; however these people use their senses (hearing, sight, touch, smell and taste) to capture additional information from the environment. Using such information, and eventually the data given by regular sensors, the HBS produce knowledge that represents the current value of a certain context variable (e.g., the emergency type or size). Although the HBS are not accurate, they represent our best choice when the observed variable is not measurable by a regular sensor.

The HBS uses a mobile device that allows him/her to share that knowledge with others and also to sense context variables (see the sensing layer in Figure 6), e.g., the presence of other responders in the area by using an opportunistic network (oppnet). As explained before, it is assumed that every component participating in this solution has a network interface that allows it to communicate with others through a digital network interface.

**Figure 7.**
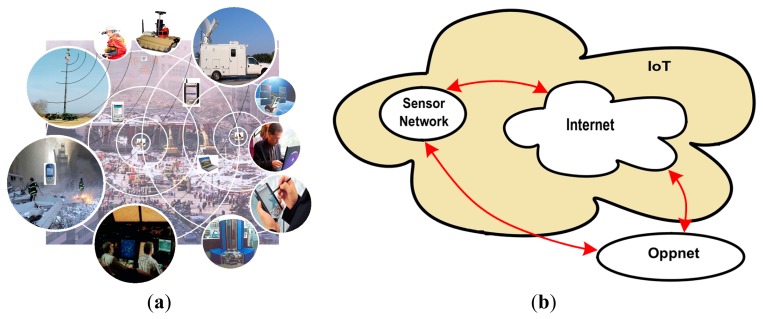
(**a**) Heterogeneous connectivity scenario; (**b**) Communication infrastructure.

The *communication layer* is responsible for providing interaction capability to components participating in the first response process. Because there is no universal network interface, this layer is implemented as a set of heterogeneous solutions, hopefully linked through communication bridges (Figure 7a).

Typically two types of communication solutions are used in these scenarios: *infrastructure-based* and *ad hoc* networks. The first one uses the regular communication infrastructure (*i.e.*, satellites, cellular towers and wired networks) and also mobile antennas (e.g., deployed in vehicles) to communicate the resources in the affected area, with remote components (e.g., emergency offices, data centers, remote experts, government agencies). We call these *communication units* (CU); components that link resources within the affected area with those that are outside the emergency area (see Figure 6). Thus, these CUs allow the remote gathering and analysis of information (e.g., through Internet) that comes from sensors deployed in the emergency area (Figure 7b). The reliability of the links provided by these networks can be estimated at the design time; therefore, the capability of a HWSN to disseminate information can be determined before implementing it.

The same occurs when ad hoc networks are used as communication support. The role of these networks is to provide and enhance the communication links in the field, increasing thus the information availability in that area and reducing the improvisation during the response process [4,14,15]. The use of opportunistic networks (*oppnet*) is highly recommended because they can work although the regular communication infrastructure is not available.

An oppnet is a peer-to-peer application-oriented mesh, able to support ad hoc interactions among stationary and mobile units that are physically close; e.g., sensors, human-based sensors and communication units deployed in the affected area. The oppnets are built in the application layer and use a “store and forward” paradigm for transmitting messages [35]. The nodes participating in these networks can act as gateways bridging oppnets and regular Internet channels, allowing thus services integration and information exchange according to the IoT paradigm.

Provided that oppnets have quite short communication thresholds, the mules available in the area help connect disjoint networks, allowing thus asynchronous communication among resources in the field. These mobile nodes are typically implemented using computing devices installed on police vehicles, fire trucks and ambulances (see the communication layer in Figure 6).

The *information persistence layer* is responsible to store and share the supporting information, allowing participants to coordinate their activities and make better and timely decisions. Several types of components can play this role, for instance the HBS, Mu and WU. Particularly, the WU can also act as an information gateway, particularly if they are accessible through the Internet.

Finally, the *application layer* is responsible for providing a direct and useful service to the end-users, e.g., first responders, incident commanders, emergency managers, government agents, hospital personnel and civilians (regular drivers). These applications make use of the services provided by the rest of the proposed architecture.

The role, behavior and services of every component type described in the architecture are clearly delimited and computable at the design time. Several emergency response agencies can take advantage of it to design not only their own response processes or the supporting system, but also the coordination of activities with other agencies, which is a recurrent limitation reported by the researchers after every medium-sized and large incidents [14,15].

The technology required to implement emergency response solutions adhering to the proposed architecture is available, and part of it is already deployed in many public spaces; *i.e.*, WiFi and GSM antennas, mobile devices with several communication capabilities, surveillance cameras, public speaker systems and displays, remotely controlled traffic lights, traffic sensors, weather sensors, and ad hoc communication and positioning services. Only considering these regular components it is possible to design solutions to make the response process to urban incidents more effective.

## 6. Application Example

In order to exemplify the usefulness of this proposal, we will analyze some aspects of the response process conducted by first response task forces on 22 February 2012, after a train crash at a central station in Buenos Aires, Argentina. After 8:30 am a train crashed with the end of the line at the *Once Station*. The accident left fifty-two dead people and over six hundred injured.

The Once Station is the third most important in Buenos Aires. It is the head of line for a very large rail network that goes to the west of the city. Only 3 km away from the city center, this station is a hub for buses, subway and other train lines. Every day one million people go through this hub for transportation connections (train-bus-subway) or as their final station. Clearly this is a key and crucial place in which emergency response processes must be planned, because the potential impact of a hazardous event is usually high. During the emergency preparedness process (*i.e.*, before an emergency happens) the first response organizations should determine the best way (in terms of time and effectiveness) to diagnose the situation, conduct a search and rescue, and assist injured people [36]. Counting on a model that allows designers to analyze possible limitations of the response plan can help increase the effectiveness of the activities involved on it.

### 6.1. Emergency Response Process

When the accident took place, the diagnosis process involved some minutes due the complexity of the physical scenario, and it began when the first firefighter company arrived to the area in question. Although the accident was recorded by various surveillance cameras (*i.e.*, a regular sensor), neither automatic alarms were activated nor video records were shared with the emergency centers that could have helped reduce the reaction time. Analyzing the accident video records (using one or more HBS) it is possible to estimate the size and type of the emergency in approximately one minute. Therefore, sharing this video with the proper emergency center could have triggered a quick response in only a few minutes resulting in more saved lives.

Once the first diagnosis was known, the emergency center performed a formal dispatch of resources, particularly firemen, police officers and ambulances. By that time, the traffic and communications in the area had collapsed, therefore the dispatched resources (many of them were HBS) experienced numerous difficulties, not only in arriving to the emergency site, but also in receiving and reporting information.

Two helicopters, 110 ambulances, 55 police vehicles (over a hundred policemen) and six firefighting companies (over three hundred firemen) participated in the response process. Fifteen hospitals received the injured people (a total of 676 people according to the official report [37]).

The ambulances started transporting these people to the three closest hospitals. Once these hospitals were overcrowded, other options were taken. However, for this situation to become visible to the ambulances was a complex task that required a considerable time. Therefore, many ambulances tried to leave injured people in more than one hospital until they found one able to receive them. The first paramedic (*i.e.*, HBS) arriving to an overcrowded hospital could record the hospital status in one or most witness units, and thus other ambulances accessing such an information can know where exactly to transport the people.

After the first 12 h, no lists of dead and injured people were available. Therefore, relatives and friends of potential victims were asking in different hospitals and at the city morgue for hours, interfering with an already complex response process. The hospital managers (*i.e.*, potential HBS) could have made public the list of injured people located there, e.g., through a Web portal or in a witness unit, allowing thus civilians (also HBS) to perform their searches quickly and without jeopardizing the emergency response activities.

The search and rescue process took all day. At the end of the day there was still a missing person, who was found 48 h later. He was a young man who got into the train 20 min before the crash. He was travelling in a compartment where passengers are not allowed to be. The rescuers scanned over the spot without noticing his presence.

This situation could have been addressed more effectively through the use of a contactless Smart card called SUBE (that stands for Electronic Ticket Unified System), that identifies each user. By making a small extension to the current ICT infrastructure supporting the SUBE, it is possible to sense (e.g., count) the number of people in the train, and also the identity and location of each passenger. If this information is shared through a witness unit, then it is possible to accelerate the response process, triggering parallel procedures that help reduce the injury. It also would have allowed an early detection of the missing person. An extra support for locating people could also be obtained using localization mechanisms based on cellular phones (with the WAP-OTA protocol).

Concerning communication support in the affected area, there is no clear information in the emergency reports, except that the telephone networks (both wired and wireless) collapsed immediately after the incident and that they were down for some hours. There is no report on the use of mobile antennas and only VHF radio systems seem to be used to support communication in the field. This situation is aligned with many other large incidents affecting urban areas [15,18,20].

The use of sensor networks, for example focused on traffic control, could help by changing the frequency of the traffic lights when an emergency vehicle is approaching, or route the regular vehicles toward safe areas where they do not interfere with the response process. Moreover, oppnet-based applications could be used to coordinate the response activities in the field and share valuable information for local decision making.

In this accident, as with many other urban emergencies, there was no predefined response plan, leading to a collapse in the first response process. A similar situation happened in the same place on 20 October 2013, and the effectiveness of the response process was similar to that previously described. However, these situations could have been addressed during a preparedness phase, using a HWSN. For instance, the emergency detection and diagnosis could be done in a few minutes using the technology already deployed in the area. This information and also the predefined emergency plan could be disseminated before the communication system collapses. The communication in the field could be addressed using oppnets, and the role of each node and information flow could be predefined. The amount and type of resources required could also be pre-calculated improving thus the time and effectiveness of the response process.

Although the technology to implement IoT-based solutions that support urban emergency responses is already available, there are no clear guidelines about the role played by each component type, how to integrate them, and how to design the behavior of an integral solution. In that sense, we hope that this proposal contributes to modeling solutions for the described and also other work scenarios. The definitions presented in Section 4 allow determining the behavior of the different types of nodes, and the interaction capabilities among them. We can therefore model the whole response system or a part of it (for instance, the search-and-rescue or the medical assistance process). Knowing what we can expect of an emergency response process or an IoT-based solution will always be beneficial, although we decide to not improve them. In that sense, the use of HWSN can make an important difference when we try to understand the potential impact of an implementable solution.

### 6.2. Design of an Emergency Response Supporting System

**Figure 8.**
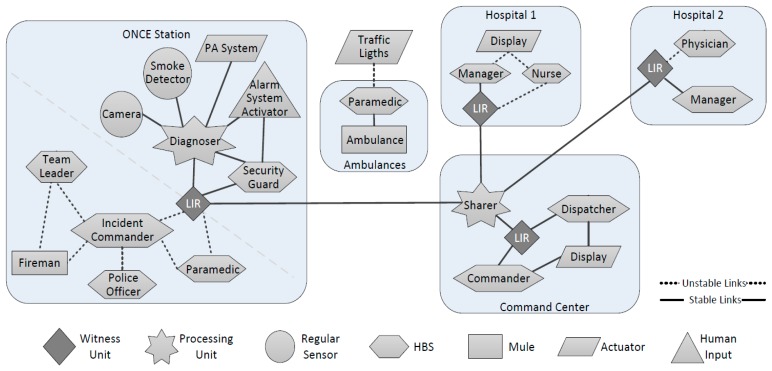
Model of a simple IoT-based system to support emergency response processes.

Figure 8 uses the nomenclature proposed in [38] to represent the design of a simple and hypothetical IoT-based system, which could be used in the described emergency in order to allow the information flow among the participants. This system can be implemented using technology that is already available in the organizations that participated in the response process.

The system design was simplified to ease its readability; however, the system model is still complex enough as to show the usefulness of the proposed modeling approach. In this case we have considered that all resources are present during the same time slot; *i.e.*, all of them are working simultaneously.

In Figure 8 we can see four islands: the affected area, the city command center, and two hospitals. We can also see ambulances transporting injured people to the hospitals, and several other node types working within the islands. Provided that the communication support collapses immediately after these events, during the preparedness phase the emergency response organizations must design a robust channel to exchange information among the main players (*i.e.*, islands). The system implements such a channel using witness units (*i.e.*, Local Information Repository—*LIR*) communicated through stable communication links. The information exchange is managed by a processing unit (*sharer* in Figure 8), which plays an ad hoc process. Such a process takes advantage of the idle bandwidth of the channel in order to replicate the key information among the several witness units supporting the process (one unit per island). The nodes working within an island consume the information from the LIRs. These repositories can be implemented using several computing devices (typically, from a laptop to large servers), depending on the amount of information that they need to manage.

Using the definitions presented in Section 4 we can determine, for instance, the probability that one HBS can feed a LIR with information when required, and also the probability that such information can be disseminated to other LIRs. In fact we can compute the HWSN represented in such a design, according to the behavior predefined for each node and link in order to determine fast information routes, slow routes and also broken routes. For instance, we can expect to have no communication between the resources in the islands and the ambulances when the later are travelling between the affected area and the hospitals and vice versa. Moreover, we can expect an intermittent communication with the ambulances when they arrive to an island.

Computing this system model we can identify its strengths and weaknesses, and improve it iteratively at the design time. Thus, we can implement the system only when its design is robust enough. Moreover, the model can also be used to determine the best procedures to follow during the emergency diagnosis, response and recovery, by making these procedures more effective and efficient.

### 6.3. Analysis of the System Design

In order to illustrate how to use these system models to determine the expected capabilities and limitations of a possible implementation, let us consider the proposal presented for the Once Station (Figure 8). There we can see two subsystems: the ICT infrastructure deployed in the train station and the infrastructure used to support the response process. The first one considers dedicated and stable links to support interactions among the *regular sensors* (*i.e.*, surveillance cameras and smoke detectors), a *human data input* (*i.e.*, the activator of the alarm system), an *actuator* (e.g., the public address system -PA system), the *processing unit* (*i.e.*, the system that diagnoses hazardous events), the *HBS* working in the train station (e.g., security guards that are part of the permanent personnel), and a *witness unit* (*i.e.*, the local information repository). The security guards and the diagnosing system can update on-demand the information shared through the LIR.

The second subsystem considers unstable links (e.g., WiFi-based networks) that support the interactions among the *HBS* (*i.e.*, the incident commander, police officers, paramedics and team leaders), *mules* (e.g., some rescuers) and the *local information repository*. The incident commander and the paramedics exchange, according to their role, the information with the LIR.

In this analysis we are going to focus only on the second subsystem, because it has more uncertainty and space for improving the information flow and availability. In order to make a more effective analysis of these features of the system design, we used an already extended version of the NS-3 simulator [39] that implements the types of nodes considered in a HWSN. We model the Once Station as a square area of 200 × 200 m, where 43 nodes were deployed: ten (firefighters) team leaders, one incident commander, ten paramedics (one per ambulance), twenty police officers (because they use small units) and two firemen acting as mules. We considered stationary nodes to represent the incident commander, police officers that isolate the affected area, and paramedics assisting people at the emergency place. The team leaders performing the search-and-rescue activities followed a RPGM mobility pattern [40] and they have medium mobility according to the guidelines given in [41]. High mobility was set for mules (firemen), who followed a predefined path to interconnect the team leaders with the incident commander.

We considered a HWSN based on WiFi (IEEE 802.11 standard) without infrastructure, particularly an oppnet. The effective communication rage of a node was set to 30 m, that is an average value that we can obtain in open areas with obstacles (like a train station), considering to have a minimum bandwidth of 50 Kbytes for information exchange.

The routing protocol used on the oppnet was Optimized Link State Routing (OLSR) [42], and all communications were performed using one or two hops. The HBS generated a message per second, which contained a piece of information of 2 Kbytes. All pieces of information were unique. Every node intended to deliver its messages through the network immediately after they were created, and the destination node was always the LIR. In these simulations we have assumed that all nodes are similar among them in term of their technical features and computing capabilities. Particularly, the node model corresponds to the features of an i-Phone 4.

Fifteen simulations were done for each scenario and eight of the most representative simulations were selected for the posterior analysis. Table 1 summarizes the simulation parameters and Table 2 indicates the parameters used for the mobility models of each node type.

**Figure 9.**
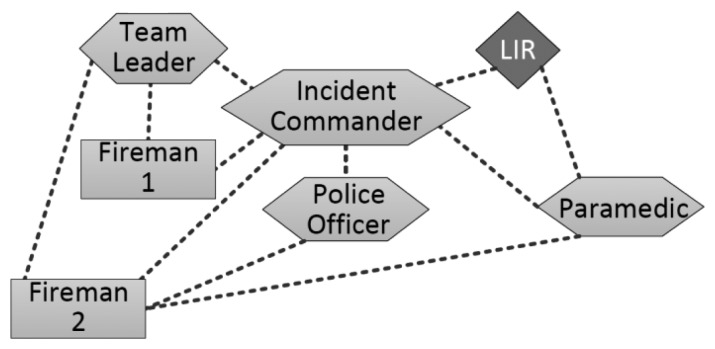
Interaction scenario 2.

**Figure 10.**
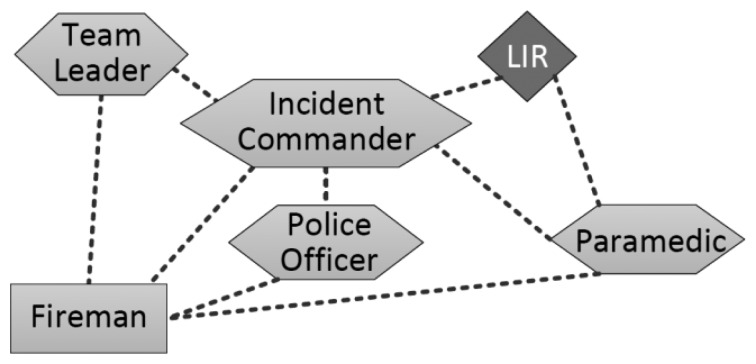
Interaction scenario 3.

Considering this general structure for the interaction space, we have defined three specific scenarios in which we have changed the path of the mules just to illustrate the impact that it has on the information availability and flow. The first specific scenario considers two mules connecting only team leaders and incident commanders (Figure 8). In the second one, a mule keeps the former path (Fireman 1 in Figure 9) and the other extends that route to allow the information flow with paramedics and police officers (Fireman 2). In the third specific scenario, both mules follow the route used by the Fireman 2 in the previous scenario (Figure 10). Moreover, in these simulations we have changed the communication range of the network links in order to illustrate how the technology used to implement these links impacts on the information flow and availability.

**Figure 11.**
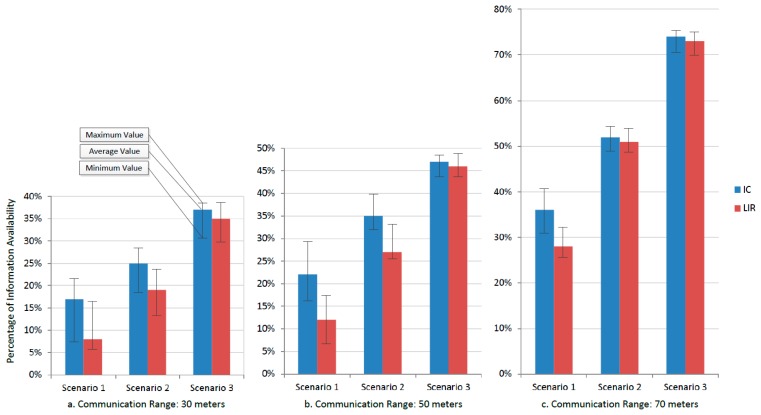
Information availability to the IC and LIR.

Provided that the Incident Commander (IC) is the main decision maker of the emergency response process, let us analyze how much information he has available to make decisions. Moreover, we have also analyzed the amount of information that the response process makes available for the LIR, considering that such a component is the only link that the system has for sharing information with other islands (*i.e.*, other response efforts being conducted in parallel way). In order to ease the visualization of results, we are not considering the legacy information available in the LIR previous to the emergency situation. Figure 11 shows the percentage of information that is visible for the IC and the LIR, considering the three mobility scenarios and the three communication ranges.

The results shown in Figure 11a indicate that, in these scenarios, the information availability increases with the number of interconnected nodes. Therefore, the use of mules plays an important role, due to the instability of the communication links available to support the response process. The tendency is similar in the three scenarios, regardless of the communication range of the nodes. However increasing the communication range increases also the information availability (Figure 11a–c), which is something that we can expect since a large communication range provides more opportunities for information exchange among the nodes.

The variability of the information availability (see maximum and minimum values in Figure 11) decreases while increases the probability of interactions among nodes. During the system design phase, the developers can use these results to support their implementation decisions. For instance, if the technology that they have available for implementing the links has short communication range, they can add mules into the system design (even with different paths) until reaching the level of information availability that they need. The number of mules that are required and also their paths can be determined using simulations like the one described above. The developers can also try several others strategies for sharing information, and then decide which model to implement based on the requirements and advantages of each alternative.

Figure 11 also shows that the information availability to the IC is higher than to the LIR. This evidences a design limitation in the interactions supported by the system. Provided that the LIR is the only witness unit in the island and the link with remote collaborators, the information availability in that node should be at least similar to the availability in the IC. Such a design limitation can be detected using the simulation results or doing a visual inspection to the system design; for instance, in Figure 8 we can see that the LIR interacts only with the IC and paramedics. This design limitation can be overcome just adding the LIR to the mules path, or increasing the nodes communication range.

Using the proposed modeling approach the designers can explore several alternatives without the need to implement them. Only when the design is stable and the properties of the system have been reached from a theoretical point of view, the system implementation will have low uncertainty and risk.

## 7. Conclusions and Future Work

Modeling IoT-based systems is a complex and still open issue. This paper proposes a modeling approach to help designers address part of these systems design and evaluation. Particularly, this modeling approach allows specifying the role of the entities participating in the solution and the interactions among them.

The roles that can be assumed by the network nodes can be formally defined by extending or specializing the existing roles. The way in which those roles can be composed or linked in a HWSN are also formally defined, therefore, every system design can be computed using a programming language or simulations, in order to determine the properties of each system model. Thus, it is possible to early evaluate several design alternatives, involving an affordable effort. These early evaluations will contribute to reducing development times, costs and risks. Following this modeling approach we can expect that the quality of the solutions to be implemented also improve.

This approach could also be used for designing more traditional wireless sensor networks. In this case the designers would just not make use of some of the node types.

The article also illustrated the use of the proposed approach through the modeling of a simple IoT-based system that supports first responders during medium-sized or large urban emergencies. The application example considers many information producers and consumers, and shows how regular sensing systems deployed in the affected areas could be used to help improve the efficiency and effectiveness of the first responses. The system is based on the Internet of Things paradigm and it is used to provide some intelligence to the response process. The usefulness of this hypothetical system is illustrated by analyzing a real first response process performed after a train crash happened at the Once train station in Buenos Aires, Argentina on 22 February 2012. The article also analyzes how this solution could have helped improve the reaction time and response activities after such an incident. Particularly, the information availability in two nodes of the system was analyzed using simulations, and some design and implementation alternatives were also evaluated to try increasing such availability.

As mentioned before, the goal of performing early evaluations to these systems designs is to be sure about the solution capabilities and limitations, before investing effort in the implementation activities. The next steps in this initiative are particularly focused on determining just how accurate are the diagnosis obtained through the analysis of these systems designs.

## Figures and Tables

**Table 1.** Simulation parameters.

**Parameter**	**Value**
Simulation time	3600 s
Simulation areas	200 × 200 m
Number of nodes	43
MAC protocol	IEEE 802.11
Propagation model	TwoRayGround
Transmission range	30 m
Routing protocol	UM-OLSR
Frequency of msg delivery	1 s
Msg weight	2 Kbytes

**Table 2.** Parameters of the mobility models for each node type.

**Parameter**	**Value**
*IC, Paramedics and Police Officers:*	

Mobility model	Stationary
Max. speed	0.1 mps
Max. pause	60 s

*Team Leaders (firemen):*	

Mobility model	RPGM
Avg. nodes per group	4
Max. speed	2 mps
Max. pause	360 s

*Mules (firemen):*	

Mobility model	Predefined Path
Max. speed	4 mps	
Max. pause	180 s
Number of stops	3
Circuit length	350 m
